# Preliminary insights into RNA in CSF of pediatric SMA patients after 6 months of nusinersen

**DOI:** 10.1186/s13062-023-00413-6

**Published:** 2023-09-13

**Authors:** M. Garofalo, S. Bonanno, S. Marcuzzo, C. Pandini, E. Scarian, F. Dragoni, R. Di Gerlando, M. Bordoni, S. Parravicini, C. Gellera, R. Masson, C. Dosi, R. Zanin, O. Pansarasa, C. Cereda, A. Berardinelli, S. Gagliardi

**Affiliations:** 1 IRCCS Mondino Foundation, Pavia, Italy; 2 Neurology IV-Neuroimmunology and Neuromuscular Diseases Unit, Fondazione IRCCS Istituto Neurologico Carlo Besta, Milan, Italy; 3 Department of Biosciences, University of Milan, Milan, Italy; 4 Department of Biology and Biotechnology, University of Pavia, Pavia, Italy; 5 Department of Brain and Behavioral Sciences, University of Pavia, Pavia, Italy; 6 Unit of Medical Genetics and Neurogenetics, Fondazione IRCCS Istituto Neurologico Carlo Besta, Milan, Italy; 7 Center of Functional Genomics and Rare Diseases, V. Buzzi Children’s Hospital, 20154 Milan, Italy

**Keywords:** Spinal muscular atrophy, Nusinersen, Cell-free RNA, Transcriptomics

## Abstract

**Background:**

Spinal muscular atrophy (SMA) is a rare autosomal-recessive neurodegenerative disorder caused by mutations in survival motor neuron 1 (*SMN1*) gene, and consequent loss of function of SMN protein, which results in progressive loss of lower motor neurons, and muscular wasting. Antisense oligonucleotide (ASO) nusinersen (Spinraza®) modulates the pre–mRNA splicing of the *SMN2* gene, allowing rebalance of biologically active SMN. It is administered intrathecally via lumbar puncture after removing an equal amount of cerebrospinal fluid (CSF). Its effect was proven beneficial and approved since 2017 for SMA treatment. Given the direct effect of nusinersen on RNA metabolism, the aim of this project was to evaluate cell-free RNA (cfRNA) in CSF of SMA patients under ASOs treatment for biomarker discovery.

**Methods:**

By RNA-sequencing approach, RNA obtained from CSF of pediatric SMA type 2 and 3 patients was processed after 6 months of nusinersen treatment, at fifth intrathecal injection (T6), and compared to baseline (T0).

**Results:**

We observed the deregulation of cfRNAs in patients at T6 and we were able to classify these RNAs into disease specific, treatment specific and treatment dependent. Moreover, we subdivided patients into “homogeneous” and “heterogeneous” according to their gene expression pattern. The “heterogeneous” group showed peculiar activation of genes coding for ribosomal components, meaning that in these patients a different molecular effect of nusinersen is observable, even if this specific molecular response was not referable to a clinical pattern.

**Conclusions:**

This study provides preliminary insights into modulation of gene expression dependent on nusinersen treatment and lays the foundation for biomarkers discovery.

**Supplementary Information:**

The online version contains supplementary material available at 10.1186/s13062-023-00413-6.

## Background

Spinal muscular atrophy (SMA) is a rare autosomal-recessive neurodegenerative disorder (incidence 1/8000 live births) characterized by progressive loss of lower motor neurons, with subsequent muscular atrophy and, in the most severe forms, early death due to respiratory failure. A wide range of SMA phenotypes has been described according to age of onset and level of motor functions achieved, ranging from in utero to adult onset SMA (type 0–4) [1]. It is caused by mutations in survival motor neuron 1 (*SMN1*) gene (locus 5q11.2-q13.3), which lead to the loss of functions of the encoded protein SMN. However, up to 10% of full-length SMN is produced by the centromeric homologous *SMN2* gene, which partially compensates the lack of *SMN1*. Hence, *SMN2* copy number has been found inversely correlated with disease severity, contributing decisively to the observed SMA clinical heterogeneity [2]. SMN is an almost ubiquitously expressed protein so the prevalent vulnerability of motor neurons to its quantity/quality defect is not completely clear. Many concurrent causes have been evoked, like the impact of the surrounding glial cells on the neuron functions, as well as the involvement of other modulating genes or modifiers factors such as microRNAs (miRNAs) or long non-coding RNA (lncRNAs) [3, 4]. Indeed, SMN seems to be involved in RNA metabolism, in particular in small nuclear ribonucleoprotein (snRNP) biogenesis, alternative splicing, trafficking of RNA-binding proteins and translation of target mRNAs in neurites [5]. In 2017, the first disease-modifying drug was approved in Europe for the treatment of SMA. Nusinersen is an antisense oligonucleotide (ASO) that modulates the pre–mRNA splicing of the *SMN2* gene, inducing the production of a greater amount of full-length, biologically active, SMN. Nusinersen shown to ameliorate motor function in pediatric [6], and adult SMA patients [7]. Nusinersen is administered intrathecally via lumbar puncture at day 0, 14, 28, 63 and then every four months, after removing an equal amount of cerebrospinal fluid (CSF). These route and plan of administration offer the unique opportunity to longitudinally analyze simultaneous CSF and serum samples in order to search for candidate biomarkers of treatment efficacy. Evidence showed decreasing levels of specific markers of neurodegeneration (Neurofilaments (NF), and Tau) in CSF of infantile SMA during nusinersen treatment [8], suggesting a recovery in motor neuron damage. On the other hand, CSF NFs fail to be a diagnostic and monitoring marker in adolescent and adult SMA type 2 and 3 patients. Beside proteins, also several types of RNA have potential use as biomarkers of disease and treatment, such as mRNA, miRNA and lncRNA [9–11]. These can be found as cell-free RNAs (cfRNAs) in extracellular compartment, such as CSF [12]. Both the collection of CSF before each nusinersen injection and the fact that this treatment involves ASOs, lead us to investigate alteration of cfRNAs is CSF of SMA patients after six months compared to baseline.

A recent published work identified miRNAs as potential biomarkers to monitor response to nusinersen in pediatric SMA patients. This study demonstrated that muscle-specific miRNA serological levels decrease over disease course upon nusinersen treatment identifying these circulating molecules as possible non-invasive biomarkers of nusinersen therapeutic effect in SMA patients [13]. In addition, miR-206 a muscle-specific miRNA and miR-9, a neuron-related miRNA, were identified as informative biomarkers in serum of animal model and SMA patients [14]. However, no data are available so far, about long RNAs, such as coding mRNA and lncRNAs in the disease. Also, at present, entry of molecular biomarkers into SMA clinical practice is still far to be realized and represents an important challenge.

In this work we will present a quantitative explorative study of mRNAs and lncRNAs analyzed by RNA-sequencing at baseline, i.e. before first injection of nusinersen (T0), and at fifth injection, after 6 months of nusinersen treatment (T6).

## Materials and methods

### Study subjects

A cohort of 18 genetically defined pediatric SMA type 2 and 3 patients followed-up at Child Neuropsychiatry Unit of IRCCS Mondino Foundation (Pavia, Italy) and at the Developmental Neurology Unit of Fondazione IRCCS Istituto Neurologico Carlo Besta (Milan, Italy), was included in the study (Table 1). All SMA patients, with confirmed diagnosis by genetic analysis that were agreed to undergo treatment, have been included. At the time of this study no SMA1 patients were followed in both Pavia and Milan Institutes. All patients received nusinersen dosages completely and no significant medical event during treatment period were observed. Clinical assessment comprised the Hammersmith Functional Motor Scale Expanded (HFMSE).

**Table 1:** Demographic data of pediatric SMA patients

	Gender		Age of onset(months)
	Female	Male	
SMA2	8	7	9.8 ± 2.3
SMA3	1	2	44.6 ± 7.5

The study was performed in accordance with the ethical standards of the Declaration of Helsinki. CSF samples were obtained by lumbar puncture, after parental written consent, right before first nusinersen infusion (T0, baseline) and after 6 months of treatment (T6). Biological samples were stored at − 80 °C until use.

### CSF storage and RNA extraction

After withdrawal, CSF samples were centrifuged at 2000 × *g* for 10 min at room temperature and immediately stored at − 80 °C. RNA was extracted from 400 µl of CSF with miRNeasy Serum/Plasma Kit (Qiagen, Germany) following manufacturer’s instructions. Then, RNA was quantified and its quality assessed with Nanodrop ND-100 spectrophotometer (Nanodrop Technologies, USA).

### Library preparation and sequencing

RNAs to be sequenced were processed with RiboCop rRNA Depletion Kit (Lexogen, Austria) starting from 50 ng of RNA. Sequencing libraries were prepared using CORALL Total RNA-Seq Kit (Lexogen, Austria) following manufacturer’s instructions and performing 20 PCR cycles. Then 1.8 pM of pooled and denatured libraries were loaded on a NextSeq 500/550 High Output Kit v2.5 (150 Cycles) (Illumina, USA).

### Bioinformatic, databases and statistical analyses

Bioinformatic analysis for obtaining differentially expressed (DE) genes and gene enrichment analyses were conducted as in Garofalo et al. [15], except a |log2(disease sample/healthy control)|≥ 0.5 was applied. Databases used for investigation of DE genes were NCBI and Ensembl. Literature searches were conducted on PubMed. The linear regression model was constructed on Prism GraphPad 8.0.2 software (GraphPad Software, USA).

## Results

### Analysis of DE cfRNAs in CSF of pediatric SMA patients after 6 months of nusinersen treatment compared to baseline

To identify cfRNAs in CSF associated with nusinersen treatment of SMA patients, we performed RNA-sequencing analysis through Next Generation Sequencing (NGS) approach. Eighteen CSF cfRNA samples obtained from all pediatric SMA patients (named “all” cohort) treated with nusinersen at T6 were compared to CSF cfRNA samples from the respective patients at T0. We detected 48 DE cfRNAs (Table 2; Additional file 1: Table S1), of which 36 mRNAs, 1 lncRNA and 11 “other biotype” RNAs (processed pseudogenes, unprocessed pseudogene and untranscribed unprocessed pseudogenes). We found 23 down-regulated and 13 up-regulated mRNAs, the only lncRNA was down-regulated, while 7 “other biotype” RNAs were up-regulated and 4 were down-regulated.

**Table 2:** Statistically significant DE cfRNAs in CSF of “all” at T6 compared to T0

	mRNA	lncRNA	Other biotype
*DE cfRNAs in “all” at T6 vs T0*			
Up-regulated	13	0	7
Down-regulated	23	1	4
Subtotal	36	1	11
Total	48		

mRNAs, lncRNAs and “other biotype” (processed pseudogenes, unprocessed pseudogene and untranscribed unprocessed pseudogenes) number is represented in terms of up-regulated transcripts, down-regulated transcripts, subtotal and total. Transcripts were considered as DE when |log2(T6/T0)| was ≥ 0.5 and a FDR was ≤ 0.1

**Fig. 1:**
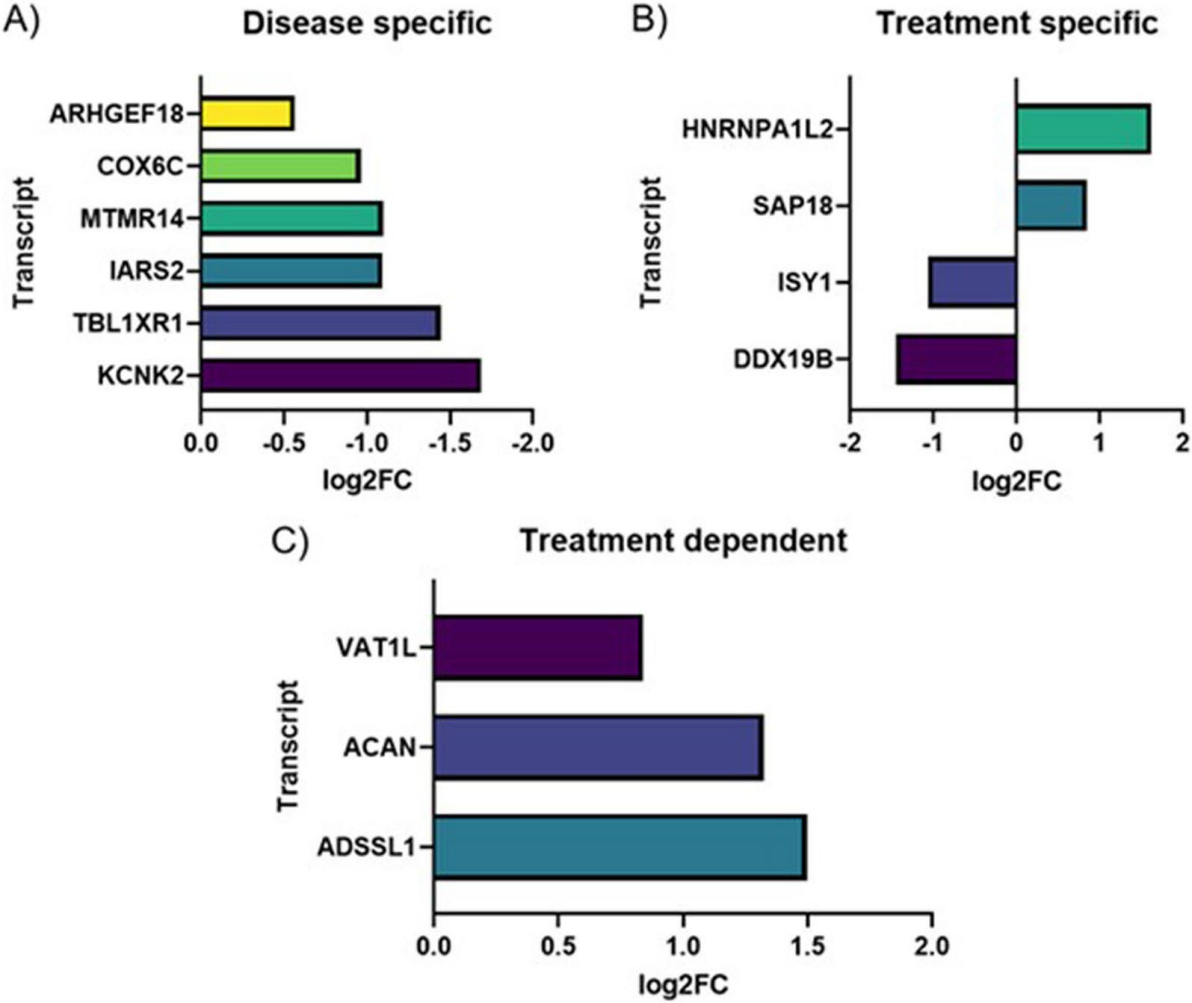
Selected DE cfRNAs in “all” subjects, subdivided into “disease specific” (**A**), “treatment specific” (**B**) and “treatment dependent” (**C**). On x-axis, the log2 Fold Change is reported, while on y-axis the corresponding DE transcript is indicated

**Fig. 2:**
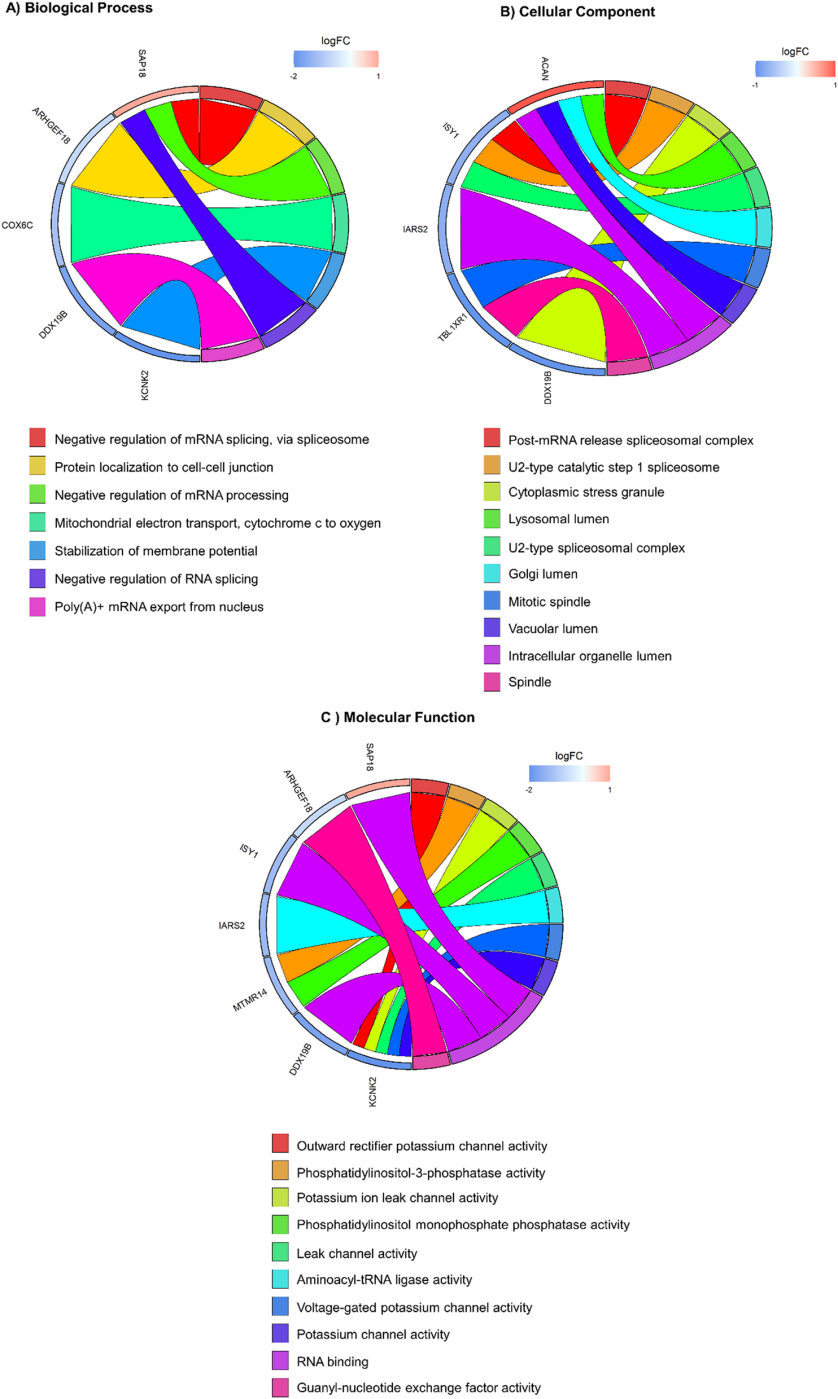
GO chord plot. Chord plot showing significantly enriched GO terms for Biological Process (**A**), Cellular Component (**B**) and Molecular Function (**C**) in “all” SMA patients at T6 versus T0 analysis. The left of the plot shows the genes contributing to the enrichment, arranged in order of their logFC (logarithmic Fold Change), which is displayed in descending intensity of red squares for the upregulated genes and blue squares for the down-regulated ones. The genes are linked to their assigned terms via colored ribbons

**Fig. 3:**
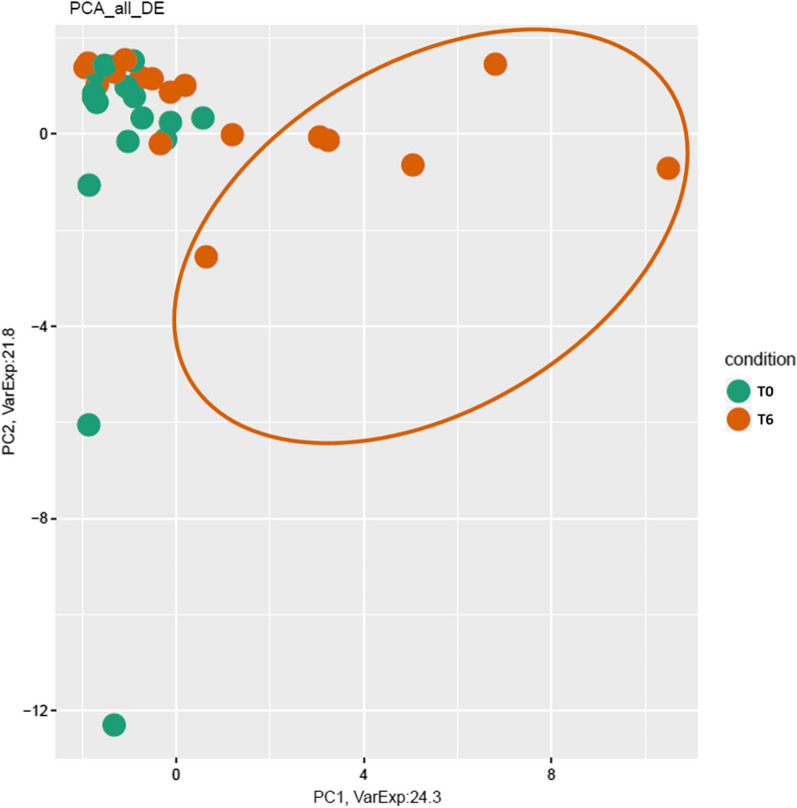
Principal component analysis (PCA) of DE cfRNAs in “all” SMA patients at T6 versus T0. PC1: 24.3 (*x-axis*) and PC2: 21.8 (*y-axis*). Samples at T0 are colored in green, while T6 samples are colored in orange. Interestingly, a subset of patients at T6 appeared to cluster less in terms of gene expression pattern. Thus, we separated these samples in the following analyses, defining them as “heterogeneous” group. Samples that did not show this pattern alteration will be described as “homogeneous”

Relying on gene databases, such as Ensembl and NCBI, and through literature search (PubMed), we divided DE cfRNAs into three categories: (1) disease specific, mostly mRNAs already involved in pathways affected in SMA pathogenesis (ARHGEF19, COX6C, MTMR14, IARS2, TBL1XR1, KCNK2) (Fig. 1A); (2) treatment specific, transcripts whose alteration may be mediated by nusinersen (HNRNPA1L2, SAP18, ISY1, DDX19B) (Fig. 1B); (3) treatment dependent, mRNA whose expression modification may be downstream nusinersen treatment (VAT1L, ACAN, ADSSL1) (Fig. 1C). We excluded all those cfRNAs not associable with SMA or nusinersen molecular effect.

### Enrichment analysis of the selected DE cfRNAs in “all” SMA patients

To show in which events were involved the selected DE cfRNAs (ARHGEF19, COX6C, MTMR14, IARS2, TBL1XR1, KCNK2, HNRNPA1L2, SAP18, ISY1, DDX19B, VAT1L, ACAN, ADSSL1), we performed an enrichment analysis of genes by Gene Ontology (GO) database. This analysis is useful for high-throughput data interpretation since it highlights biological phenomena that may be represented by DE genes [16]. Practically, a list of genes is queried, in this case the DE cfRNAs were used, and information about Biological Process, Cellular Component and Molecular Function, where given genes are involved, are provided together with statistical analyses that allow to rank the results.

We queried Biological Process, Cellular Component and Molecular Function (Fig. 2A–C). From GO Biological Process, we found significant results for SAP18, ARHGEF19, COX6C, DDX19B, KCNK2. In particular, SAP18, that we found down-regulated at T6, is involved in “negative regulation of mRNA splicing, via spliceosome”, “negative regulation of mRNA processing” and “negative regulation of RNA splicing” (Fig. 2A). GO Cellular Component query provided significant results for ACAN, ISY1, IARS2, TBL1XR1 and DDX19B. Interestingly, ISY1, that was down-regulated in our cohort after treatment, resulted as a component of “post-mRNA release spliceosomal complex”, “U2-type catalytic step 1 spliceosome” and “U2-type spliceosomal complex” (Fig. 2B). Regarding GO Molecular Function, significant results were obtained for SAP18, ARHGEF19, ISY1, IARS2, MTMR14, DDX19B, KCNK2, of whom, SAP18, ISY1 and DDX19B were related to the term “RNA binding”, and KCNK2 was involved in “outward rectifier potassium channel activity”, “potassium ion leak channel activity”, “leak channel activity”, “voltage-gated potassium channel activity” and “potassium channel activity” (Fig. 2C).

### Identification of distinct transcriptomic clusters in SMA patients after 6 months of nusinersen-treatment

We have realized a Principal Component Analysis (PCA) of all DE cfRNAs in SMA patients at T6 versus T0 (Fig. 3). From this analysis, we have visualized the distribution of each patients’ transcriptome at both time-points. Interestingly, we noticed that a subset of patients (*n* = 6) clustered differently from the whole group. Thus, we splitted the analysis by evaluating DE cfRNAs separately for the two subgroups of patients. We defined as “heterogeneous” those who appeared to be transcriptionally different from the T0 (circled in orange in Fig. 3), and as “homogeneous”, those who did not transcriptionally differ from the rest of the samples. In the heterogeneous group, we found a total of 15 DE cfRNAs, mostly mRNAs (N = 14) and up-regulated (Table 3; Additional file 1: Table S2). In the homogeneous group we found 9 DE cfRNAs, mostly up-regulated mRNAs (N = 7) (Table 3; Additional file 1: Table S3).

**Table 3:** Statistically significant DE cfRNAs in CSF of “heterogeneous” SMA patients at T6 compared to T0, and of “homogeneous” SMA patients at T6 compared to T0

	mRNA	lncRNA	Other biotype
*“Heterogeneous” T6 vs T0*			
Up-regulated	13	0	1
Down-regulated	1	0	0
Subtotal	14	0	1
Total	15		
*“Homogeneous” T6 vs T0*			
Up-regulated	7	0	1
Down-regulated	1	0	0
Subtotal	8	0	1
Total	9		

mRNAs, lncRNAs and “other biotype” (processed pseudogenes, unprocessed pseudogene and untranscribed unprocessed pseudogenes) number is represented in terms of up-regulated transcripts, down-regulated transcripts, subtotal and total. Transcripts were considered as DE when |log2(T6/T0)| was ≥ 0.5 and a FDR was ≤ 0.1

**Fig. 4:**
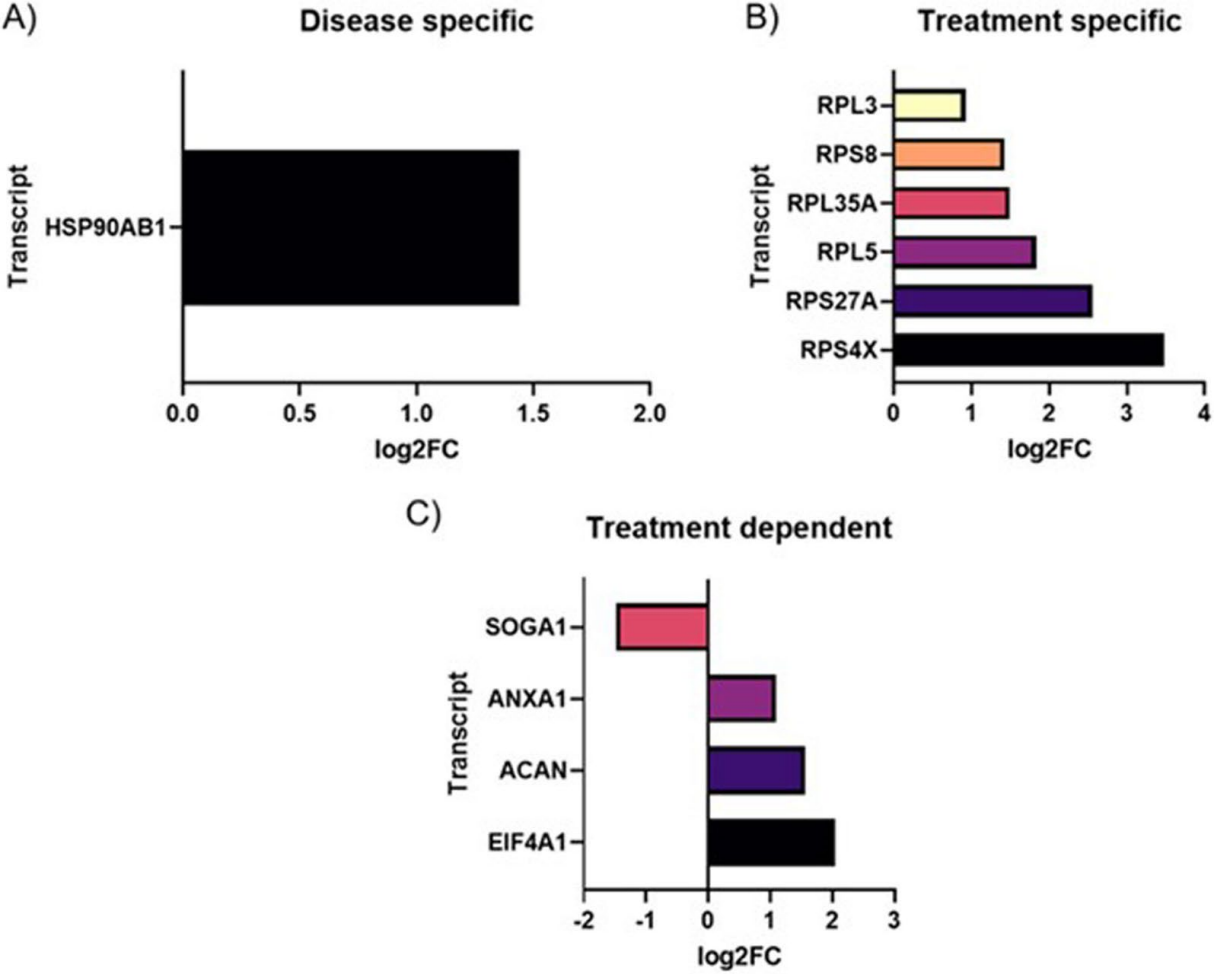
Selected DE cfRNAs in SMA “heterogeneous” and “homogenous” patients, subdivided into “disease specific” (**A**), “treatment specific” (**B**) and “treatment dependent” (**C**) for the “heterogeneous” group, and reported in the same graph for “homogeneous” group (“disease specific” in black and “treatment specific” in red. On *x-axis*, the log2 Fold Change is reported, while on *y-axis* the corresponding DE transcript is indicated

Further, we conducted a gene database/literature search for evaluating whether the DE cfRNAs in these two subgroups were disease specific, treatment specific or treatment dependent. For the “heterogeneous”, we reported this classification in Fig. 4A–C: HSP90AB1 was recognized as disease specific gene (Fig. 4A); RPL3, RPS8, RPL35A, RPL5, RPS27A and RPS4X were pertaining to the treatment specific gene group (Fig. 4B); SOGA1, ANXA1, ACAN and EIF4A1 were treatment dependent genes (Fig. 4C). Conversely, for the “homogeneous” we found only one disease specific cfRNA, IARS2 and one treatment specific MIB1 (Additional file 1: Table S3). Again, all those cfRNAs not associable with SMA or nusinersen molecular effect were excluded.

### Enrichment analysis of the selected DE cfRNAs in SMA heterogeneous patients

**Fig. 5:**
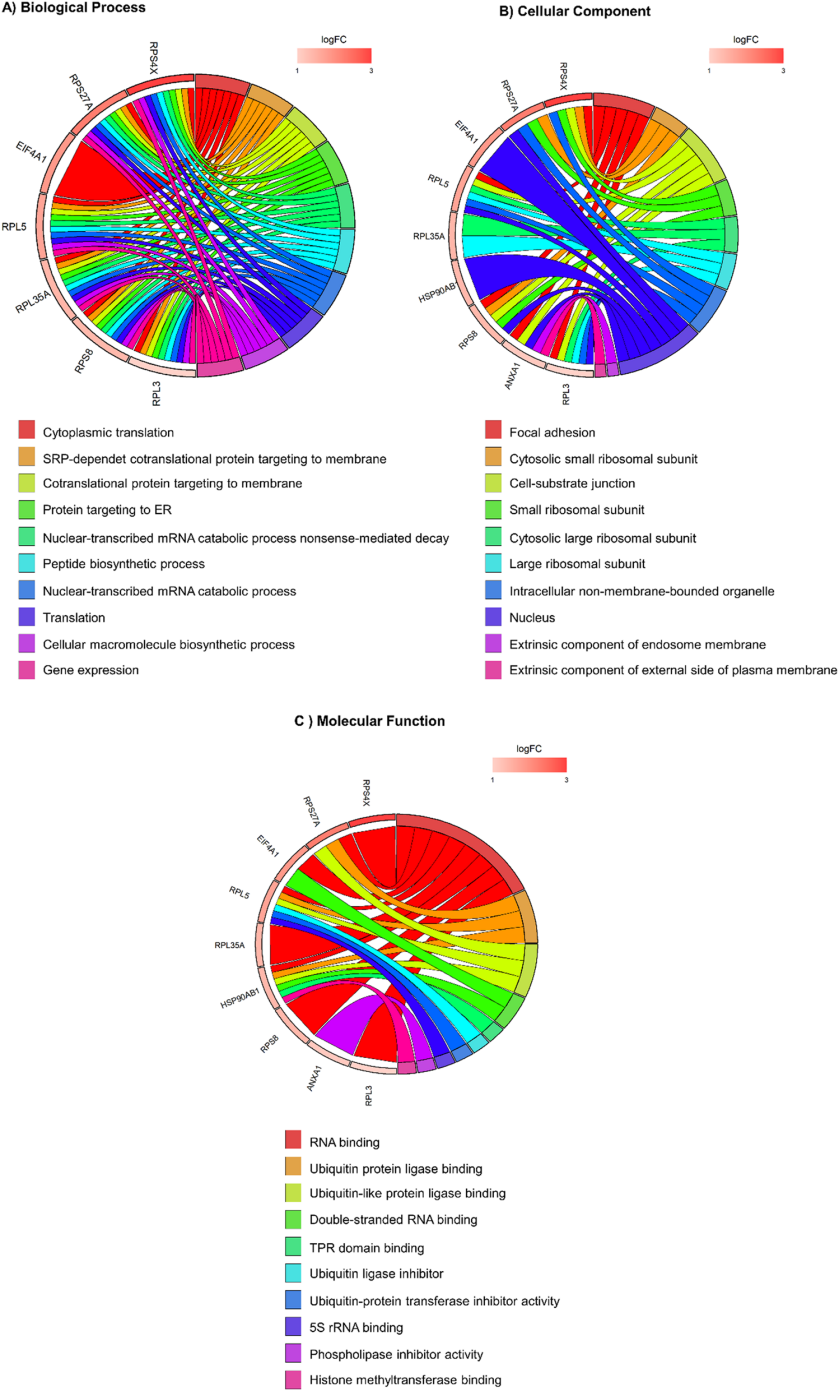
GO chord plot. Chord plot showing significantly enriched GO terms for biological process (**A**), cellular component (**B**) and molecular function (**C**) in “heterogeneous” subjects at T6 versus T0. The left of the plot shows the genes contributing to the enrichment, arranged in order of their logFC (logarithmic Fold Change), which is displayed in descending intensity of red squares for the upregulated genes, and blue squares for the down-regulated ones. The genes are linked to their assigned terms via colored ribbons

To show in which events were involved the DE cfRNAs we found in the “heterogeneous” group, we used GO database and showed enriched terms for Biological Process, Cellular Component and Molecular Function (Fig. 5A–C). From GO Biological Process, it appears in the GO chord that almost all DE cfRNAs (RPS4X, RPS27A, EIF4A1, RPL5, RPL35A, RPS8, RPL3) belong to all the terms, mostly related to mRNA transcription and protein translation (Fig. 5A). Concerning GO Cellular Component, significantly enriched terms, represented by deregulation RPS4X, RPS27A, EIF4A1, RPL5, RPL35A, HSP90AB, RPS8, ANXA1 and RPL3, are mostly related to “nucleus” and ribosomal subunits (Fig. 5B). The same genes also represented significant enriched terms for GO Molecular Function, mainly for “RNA binding”. Given the very low number (N = 2) of relevant DE cfRNAs in “homogeneous” group, the enrichment analysis was not conducted for these patients.

### Comparison of the DE cfRNAs in all analysis

**Fig. 6:**
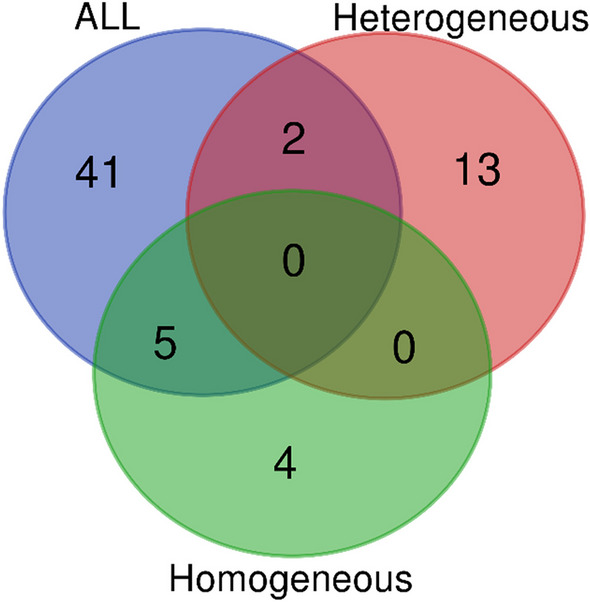
Venn diagram. Differentially expressed cfRNAs in common between the three conducted analyses (“all”, “heterogeneous” and “homogeneous” group at T6 vs T0). Two cfRNAs were common to the “all” and the “heterogeneous” group, and five cfRNAs were shared among the “all” and “homogeneous” groups

Eventually, DE cfRNAs emerged from each analysis were compared. No cfRNAs were commonly DE in the three approaches. Two DE cfRNAs were common in the “all” and in the “heterogeneous” groups, while 5 DE cfRNAs were shared among the “all” and the “homogeneous” group (Fig. 6; Additional file 1: Table S4). In Table 4, common cfRNAs with corresponding Fold Change were reported. Both DE cfRNAs common to the “all” and “heterogeneous” groups showed a higher Fold Change when patients were splitted. Moreover, the fact that 5 over a total of 9 DE cfRNA in the “homogeneous” group were in common with the analysis obtained from “all”, while in the “heterogeneous” were only 2 over 15, reflects the specificity of the latter’s gene expression pattern.

**Table 4:** Common DE cfRNAs in “all” and “heterogeneous”, and in “all” and “homogeneous”

Gene	FC “all”	FC “heterogeneous”
*Common in “all” and “heterogeneous”*		
ACAN	1.32	1.57
GNAS	1.08	2.33

Gene	FC “all”	FC “homogeneous”
*Common in “all” and “homogeneous”*		
IARS	− 1.09	− 0.96
MRC2	− 0.91	− 1.02
MUC1	1.25	1.06
PREX2	− 1.16	− 1.40
SYNE2	− 1.54	− 2.03

Each gene is reported together with the corresponding Fold Change (FC) for each analyzed group

### Genotype–phenotype correlation

We investigated a possible correlation between transcriptomic patients’ stratification and their clinical features. In Table 5 we reported SMN2 copy number, age of onset and age at baseline and the clinical change after nusinersen treatment (T6) in term of motor functions measured by the HFMSE score for each patient.

**Table 5:** Demographic, genetic and clinical feature in SMA cohort classified as “homogeneous” and heterogeneous “groups

	Homogeneous group	Heterogeneous group
ΔHFMSE	+ 2.1	+ 2.6
Age at onset (months)	15.41 ± 13.4	16 ± 15.7
Age at baseline (years)	5.3 ± 3.7	5.0 ± 3.2
SMA2	91%	83%
SMA3	9%	17%

By constructing a linear regression model, we found no correlation in terms of age at onset, age at T0, type of SMA, SMN2 copy number and HFMSE scale (Table 6).

**Table 6:** Genotype–phenotype correlation analysis

Patients	Gender	SMA type	SMN2 copy number	Age at onset (months)	Age at baseline (years)	HFMSE at baseline (T0)	HFMSE after treatment (T6)	ΔHFMSE
*Homogeneous*								
Patient 1	F	2	3	9	6	12	14	2
Patient 2	M	2	3	10	3	20	20	0
Patient 3	M	2	3	10	8	13	11	− 2
Patient 4	M	2	3	13	7	14	15	1
Patient 5	F	2	3	6	4	22	32	10
Patient 6	M	2	3	12	2	9	13	4
Patient 7	F	2	3	14	2	30	33	3
Patient 8	F	3	2	6	6	3	2	− 1
Patient 9	F	3	4	36	9	62	63	1
Patient 10	F	2	3	10	1	14	17	3
Patient 11	M	2	3	9	2	4	8	4
Patient 12	M	3	4	50	14	62	63	1
*Heterogeneous*								
Patient 13	F	2	3	9	8	19	21	2
Patient 14	F	2	3	8	6	8	9	1
Patient 15	M	3	2	48	4	62	63	1
Patient 16	M	2	3	10	1	11	13	2
Patient 17	M	2	na	9	9	7	9	2
Patient 18	F	2	3	12	2	27	35	8

## Discussion

We investigated modification of cfRNAs expression in CSF of type 2 and 3 pediatric SMA patients after five nusinersen injections (T6, 6 months), compared to first injection (T0, baseline). Our aim was to observe how nusinersen affects gene expression and whether cfRNAs in CSF, which is removed before each treatment injection, may be addressed as prognostic biomarkers. We found 48 DE cfRNAs, mostly down-regulated mRNAs. After consulting gene databases (such as Ensembl and NCBI) and the literature (PubMed), we selected DE mRNAs directly associated with disease (disease specific), directly associated to nusinersen treatment (treatment specific) and those modulated in response to nusinersen treatment (treatment dependent). We addressed ARHGEF19, COX6C, MTMR14, TBL1XR1, KCNK2 and IARS2 as treatment specific ones. ARHGEF19 (Rho/Rac Guanine Nucleotide Exchange Factor 18) belongs to the Rho GTPase GEF family, that is implicated in regulation of axon formation, growth, guidance and branching [17, 18]. Lower level of Rho GTPase inhibition in SMA patients was highlighted together with an increase of methylation at one CpG site in the *ARHGAP22,* another Rho GTPase encoding gene [19]. Moreover, GTPase genes are well known to be implicated in skeletal muscle development and regeneration [18]. The Rho family of GTPases are critical for skeletal muscle differentiation and can regulate the expression of MyoD and myogenin which dysregulation is associated to the muscle weakness observed in SMA. Deficiency of COX6C (Cytochrome C Oxidase Subunit 6C), terminal enzyme of the mitochondrial respiratory chain, was observed in a SMA-resembling case [20]. This case study highlighted the worth to screen patients with SMA clinical features for a mitochondrial disorder, when no *SMN1* mutation is present. We found interesting the down-regulation of MTMR14 (Myotubularin Related Protein 14), since in a case of type 0 SMA, negative myotubularin immunohistochemical staining was showed, suggesting the dependence of this family of proteins to SMN [21]. TBL1XR1 (Transducin Beta Like 1 X-Linked Receptor 1) is thought to be a component of histone deacetylase 3 (HDAC3) complex. HDACs modulation was addressed as a potential contributor to *SMN2* promoter activation [22] and small molecules inhibiting HDACs expression underwent clinical trials, however lacking efficacy [23]. KCNK2 (Potassium Two Pore Domain Channel Subfamily K Member 2) encodes for a component of the two-pore-domain background potassium channel protein family. Impairment of potassium and calcium channels of motor neurons have been observed in vitro as a consequence of *SMN2* expression reduction [24]. Regarding the disease specific genes that we detected, they were all down-regulated and we speculate that the reduction of their expression in CSF may be indicator of degeneration processes that are not targeted, neither indirectly, by nusinersen treatment. Regarding treatment dependent mRNAs, we identified HNRNPA1L2, SAP18, ISY1, DDX19B. Particularly, HNRNPA1L2 (Heterogeneous Nuclear Ribonucleoprotein A1 Like 2) is involved in RNA splicing, mRNA processing; and mRNA transport, thus strictly related to the effect of nusinersen and *SMN2* as we observed it up-regulated. Moreover, we postulated a link between HNRNPA1L2 and IARS2 (Isoleucyl-TRNA Synthetase 2, Mitochondrial) that was classified as disease specific. Indeed, during mitochondrial stress, a feature of SMA molecular pathogenesis, tRNAs are released from the mitochondrial matrix to the cytosol, where they can interact with hnRNPA1 protein in the cytosol [25]. Also, ISY1 (ISY1 Splicing Factor Homolog) is involved in mRNA splicing, specifically in maintaining splicing accuracy. The error rate of exon pairing is increased by the reduction of SMN1, essential for spliceosomal components assembly [26]. Being ISY1 down-regulated at T6, we hypothesized that SMN restoration through ASOs reduces splicing error rate, therefore decreasing ISY1 demand. Furthermore, DDX19B (DEAD-Box Helicase 19B) takes part to nuclear and mitochondrial splicing, and spliceosome assembly, its down-regulation may be explained by nusinersen treatment pushing to recovery of splicing machinery. SAP18 (Sin3A Associated Protein 18) is a component of the histone deacetylase complex and of the SIN3-repressing complex. One of its role is to modulate splicing regulation via its ubiquitin-like fold [27]. VAT1L, ACAN, ADSSL1 are mRNAs that were up-regulated at T6 might be activated as an indirect consequence of nusinersen treatment. Indeed, VAT1L (Vesicle Amine Transport 1 Like) enables oxidoreductase and zinc ion binding activity. Since SMN complex is known to be inactivated by oxidative stress [28], the up-regulation of this gene after six months of nusinersen treatment may reflect a protective action of VAT1L exerted as a consequence of SMN-mediated processes. ACAN (Aggrecan) is an integral part of the extracellular matrix in cartilaginous tissue. Since impairment of cartilage formation, causing aberrant bone development, has been recently described as an early event in SMA pathogenesis [29], we contextualize ACAN up-regulation as a consequence of treatment indirectly restoring molecular mechanisms impaired at disease onset. Finally, ADSSL1 (Adenylosuccinate Synthase 1) was found under-expressed in the muscle of SMA type 3 patients [30], while we found an overexpression of this gene in the CSF of our cohort of treated patients.

After performing a PCA of DE cfRNAs at T6 compared to T0, we observed that a group of patients clustered uniformly differing from all the other patients, thus we defined this group as “homogeneous”, in contrast with the group of patients that did not show this separation that we defined “heterogeneous”. To dissect the difference between these two groups, we splitted the analysis and we evaluated DE cfRNAs in the two clusters at T6 compared to the respective T0. We found interesting results for “heterogeneous” group, where only one disease specific gene was identified, HSP90AB1 (Heat Shock Protein 90 Alpha Family Class B Member 1). HSP90 inhibitors were indeed tested in a preclinical model of SMA showing amelioration of motor neuron degeneration, due to the enhanced proteasomal degradation of the Hsp90 client proteins [31].

Further, all mRNAs within the “heterogeneous” group that we classified as treatment specific, RPL3, RPS8, RPL35A, RPL5, RPS27A and RPS4X, code for ribosomal subunits, demonstrating a strong correlation with nusinersen treatment, given the involvement of SMN in ribosome biology [32]. Among treatment dependent genes, we found ACAN, already mentioned above in the “all” SMA patients’ analysis. Other noteworthy treatment dependent mRNAs pertaining to the “heterogeneous” group are SOGA1, ANXA1 and EIF4A1. SOGA1 (Suppressor of Glucose, Autophagy Associated 1) is an autophagy suppressor and this is of relevance since myofiber degeneration is associated with complete inhibition of autophagosome formation [33]. Since SMN protein levels are also regulated by autophagy modulators [33], we hypothesize that the down-regulation of this suppressor might reflect autophagy restoration. ANXA1 (Annexin A1) has anti-inflammatory activity and, given that neuroinflammation takes part in SMA pathogenesis [34], the up-regulation of ANXA1 may be a protective consequence of nusinersen treatment. Instead, regarding the “homogeneous” group, we highlighted two relevant DE cfRNAs, such IARS2 (already discussed for “all” analysis) as disease specific, and MIB1 (MIB E3 Ubiquitin Protein Ligase 1) as treatment specific.

Correlation analysis between RNA profile in the two groups and clinical data has been run but we found no associations. A limitation in this instance might be the relatively small number of patients to perform clinically meaningful stratifications. On the other hand, we correlated only clinical features that may be quantified, this approach excludes all clinical observations that are not captured by the functional motor scoring such as subjective motor improvement or response to fatigue. Also, we cannot exclude that clinical correlations may be found after a longer treatment follow-up. We hypothesized that the nusinersen effect is not yet evident in peripheral tissue as CSF after only 6 months. We plan to measure the identified markers at next infusion time point to check their expression. In particular, the spontaneous division of SMA patients in two groups may be understood first by increasing the number of samples to confirm this clusterization and also by increasing the time points may studies to figure out if this separation is maintained and if it is related to other clinical features.

Hence, our results point the way for more extensive investigations about cfRNAs role as biomarkers of nusinersen effect, and highlight the implication of the discussed molecular pathways in SMA pathogenesis, and their modulation after *SMN2*-targeting.

## Conclusions

We identified specific cfRNAs that may be used to monitor the early effect of ASOs treatment at a molecular level. Moreover, we distinguished a group of patients, named “heterogeneous”, whose transcriptomic signature differed from the group of patients that uniformly clustered as the results of PCA analysis, and also when compared to the analysis where all patients were considered. By examining in depth, the “heterogeneous” group, we realized that DE cfRNAs were highly associable to nusinersen treatment. These observations made us speculate that this group is the most responsive to the treatment at molecular levels, despite transcriptomic observations were not clearly transferable to clinical levels, possibly because the small number of patients and the short-term evaluation. Thus, based on the current findings we aim to extend our investigation by testing CSF samples in a wider cohort and at successive injections to further characterize and corroborate the potential role of these molecules in SMA longitudinal assessment.

### Supplementary Information

**Additional file 1**. File excel composed by 4 sheets. **Table S1.** All DE. Differentially expressed cfRNAs in “all” SMA patients at T4 versus T0 nusinersen injection. Gene name, gene biotype and measured log2FC are reported for each transcript. Only transcripts with |log2(T4/T0l)|≥ 0.5 and a FDR ≤ 0.1 are shown. **Table S2.** Heterogeneous DE. Differentially expressed cfRNAs in “heterogeneous” SMA patients at T4 versus T0 nusinersen injection. Gene name, gene biotype and measured log2FC are reported for each transcript. Only transcripts with |log2(T4/T0l)|≥ 0.5 and a FDR ≤ 0.1 are sown. **Table S3.** Homogeneous DE. Differentially expressed cfRNAs in "homogeneous" SMA patients at T4 versus T0 nusinersen injection. Gene name, gene biotype and measured log2FC are reported for each transcript. Only transcripts with |log2(T4/T0l)|≥ 0.5 and a FDR ≤ 0.1 are shown. **Table S4.** Venn Results. Venn analysis of DE cfRNAs in “all”, “heterogeneous” and “homogeneous” groups.

## Data Availability

The dataset supporting the conclusions of this article is available in the GEO NCBI (Entry Number GSE221900) repository.
